# Long-term effect of stents eluting 6-mercaptopurine in porcine coronary arteries

**DOI:** 10.1186/s12952-016-0063-y

**Published:** 2016-12-05

**Authors:** Matthijs S. Ruiter, Albert Doornbos, Vivian de Waard, Robbert J. de Winter, Nico J. M. Attevelt, Rob Steendam, Carlie J. M. de Vries

**Affiliations:** 1 Department of Medical Biochemistry, Academic Medical Center, University of Amsterdam, Meibergdreef 15, 1105 AZ Amsterdam, The Netherlands; 2 InnoCore Pharmaceuticals, Groningen, The Netherlands; 3 Department of Cardiology, Academic Medical Center, University of Amsterdam, Amsterdam, The Netherlands; 4 Central Laboratory Animal Research Facility, Faculty of Veterinary Medicine, University of Utrecht, Utrecht, The Netherlands; 5 Present address: Unit of Tissue Engineering, Monzino Cardiologic Center, Milan, Italy

## Abstract

**Background:**

Drug-eluting stents (DES) have dramatically reduced restenosis rates compared to bare metal stents and are widely used in coronary artery angioplasty. The anti-proliferative nature of the drugs reduces smooth muscle cell (SMC) proliferation effectively, but unfortunately also negatively affects endothelialization of stent struts, necessitating prolonged dual anti-platelet therapy. Cell-type specific therapy may prevent this complication, giving rise to safer stents that do not require additional medication. 6-Mercaptopurine (6-MP) is a drug with demonstrated cell-type specific effects on vascular cells both in vitro and in vivo, inhibiting proliferation of SMCs while promoting survival of endothelial cells. In rabbits, we demonstrated that DES locally releasing 6-MP during 4 weeks reduced in-stent stenosis by inhibiting SMC proliferation and reducing inflammation, without negatively affecting endothelialization of the stent surface. The aim of the present study was to investigate whether 6-MP-eluting stents are similarly effective in preventing stenosis in porcine coronary arteries after 3 months, in order to assess the eligibility for human application.

**Methods:**

6-MP-eluting and polymer-only control stents (both *n* = 7) were implanted in porcine coronary arteries after local balloon injury to assess the effect of 6-MP on vascular lesion formation. Three months after implantation, stented coronary arteries were harvested and analyzed.

**Results:**

Morphometric analyses revealed that stents were implanted reproducibly and with limited injury to the vessel wall. Unexpectedly, both in-stent stenosis (6-MP: 41.1 ± 10.3 %; control: 29.6 ± 5.9 %) and inflammation (6-MP: 2.14 ± 0.51; control: 1.43 ± 0.45) were similar between the groups after 3 months.

**Conclusion:**

In conclusion, although 6-MP was previously found to potently inhibit SMC proliferation, reduce inflammation and promote endothelial cell survival, thereby effectively reducing in-stent restenosis in rabbits, stents containing 300 μg 6-MP did not reduce stenosis and inflammation in porcine coronary arteries.

## Background

Coronary heart disease is a pervasive health problem and affects life expectancy worldwide. Percutaneous coronary intervention has proven a widely successful treatment to restore perfusion to the heart and is now one of the most common medical interventions [1]. In order to maintain artery patency, stents are applied in 90 % of interventional procedures [2]. Drug-eluting stents (DES) have significantly reduced restenosis rates compared to bare metal stents. DES that elute paclitaxel, sirolimus or second-generation –limus analogues such as everolimus, zotarolimus and biolimus, have been found to efficiently inhibit restenosis, reducing it to below 5 % [2]. These drugs are anti-proliferative regardless of cell type, thereby effectively reducing smooth muscle cell (SMC) proliferation, yet negatively affecting endothelialization of stent struts [3]. Uncovered stent struts are the substrate for late and very late stent thrombosis, a potentially lethal effect which necessitates prolonged dual anti-platelet therapy [4]. Premature anti-platelet therapy discontinuation is associated with mortality and major adverse cardiac events in both first- and second-generation DES [5]. Stents loaded with a drug having a cell-type specific mechanism of action may effectively inhibit SMC proliferation and reduce restenosis without negatively interfering in the process of re-endothelialization of the stented artery segment, giving rise to safer stents and lower risk of the occurrence of thrombotic events.

Nuclear receptor Nur77 (also referred to as NR4A1, TR3, NGFI-B or NAK-1), an orphan nuclear receptor of the NR4A subfamily, is involved in cellular processes such as proliferation, differentiation and migration. Nur77 has various protective functions in vascular cells both in vitro and in vivo, and exerts its beneficial effects in a cell-type specific fashion [6]. Firstly, Nur77 prevents SMC proliferation in vitro and induces a more quiescent SMC phenotype in vivo [7, 8]. In addition, activation of Nur77 promotes survival of endothelial cells and capillary sprouting [9–11]. Furthermore, Nur77 is involved in differentiation of bone marrow-derived patrolling monocytes and reduces the inflammatory response of macrophages [12–14]. Together, these functions protect against neointima formation and atherosclerosis in vivo in mouse models [15, 16]. Based on this knowledge we hypothesize that targeting Nur77 is an interesting approach to prevent in-stent restenosis, while promoting re-endothelialization and reducing local inflammation and thrombosis. 6-Mercaptopurine (6-MP) is a well-documented activator of Nur77, with demonstrated beneficial effects on vascular cells both in vitro and in vivo [17]. In a study recently published by our group, we investigated the efficacy of stents eluting 6-MP in rabbit iliac arteries [18]. We demonstrated that stents releasing 6-MP during 4 weeks according to first-order kinetics from biodegradable coatings composed of urethane-linked multi-block copolymers reduced in-stent stenosis by inhibiting SMC proliferation and reducing inflammation, without negatively affecting endothelialization of the stent surface [18]. The aim of the present study is to investigate whether 6-MP-eluting stents are similarly effective in preventing stenosis in porcine coronary arteries, as a next step towards human application. Therefore, stents were implanted in porcine coronary arteries and the stented vessels were evaluated after 3 months.

## Methods

### Stent coating

Polymer-only and 6-MP-eluting stents containing 300 μg 6-MP were prepared as described before [18]. In brief, Kaon 3.0×15 mm balloon expandable cobalt chromium stents (Fortimedix, Nuth, the Netherlands) were abluminally spray-coated with a solution of a blend of SynBiosys GLL, a multiblock copolymer consisting of 50 % w/w of poly(DL-lactide-co-glycolide) and 50 % w/w of poly(DL-lactide) and SynBiosys GPCGL, a multiblock copolymer consisting of 15 % w/w of poly(glycolide-co-PEG600-co-ε-caprolactone) and 85 % w/w of poly(DL-lactide-co-glycolide) (InnoCore Pharmaceuticals, Groningen, the Netherlands) containing 0 or 33 wt.% 6-MP (purity >99.5 %, Acros Organics). Coated stents were crimped on stent delivery systems (Clearstream DAC135 balloon catheter, Clearstream, Moyne Upper, Ireland) and sterilized by E-beam (25 kGy) by Synergy Health, Radeberg, Germany prior to implantation. Coating quality was examined visually and by scanning electron microscopy. Elution of 6-MP from coated stents was measured in vitro in 5 mL PBS buffer pH 7.4 at 37 °C (shaking water bath). Samples were collected at predetermined timepoints and refreshed with fresh buffer. The concentration of 6-MP in elution samples was measured by HPLC as described before [18]. The concentration of 300 μg 6-MP was chosen, as this was shown to be effective in reducing in-stent stenosis in rabbit iliac arteries [18].

### Animal model

In this study, 6 female Landrace pigs weighing 40–50 kg were included in the study. The animals received standard care, were housed together, maintained on a regular chow diet and were given access to drinking water ad libitum. Ten days before surgery, heart stabilization started with administration of amiodaron (800 mg/day). After surgery, amiodaron administration was continued with a lower dose (400 mg) and continued until the end of the experiment. Five days before surgery, anti-coagulation therapy was initiated by single administration of clopidogrel (Plavix, 225 mg) and aspirin (Ascal 100 mg), followed by daily oral administration throughout the entire procedure (Plavix 75 mg/day, Ascal 100 mg/day).

### Surgical procedure

Animals were anesthetized with injections of ketamine (13 mg/kg), midazolam (0.7 mg/kg) sufentanyl (0.0075 mg/kg) and propofol (3 mg/kg). Amiodaron (150 mg iv) was administered once. Prophylactic antibiotics (Amoxycillin/clavulanic acid 500/50, 10 mg/kg iv) were administered before and 1 day after the operation. Metoproprolol (3 mg iv) was administered if heart rate exceeded 80 bpm. Local analgesia at the site of entry consisted of intracutaneous injection of lidocaine (2 %) and bupivacaine (0.5 %) 1:1. Operations were performed under sterile conditions. The common carotid artery was surgically exposed and accessed with a 7 F introducer sheath (Cordis, Miami Lakes, Fl, USA) after heparin administration (100 IU/kg iv). A 7 F guiding catheter (Mach 1, Boston Scientific, Marlborough, MA, USA) was positioned in the left main or right coronary artery under fluoroscopic guidance, while injecting contrast agent (Hexabrix, 320 mg I/ml) diluted 2:1 with saline. Two or three stents were implanted in the right coronary artery (RCA), the left circumflex (LCX) and/or the left anterior descending (LAD), depending on the animal-specific anatomy. Stents were randomly assigned to arteries, with different stent types within the animal. Before stent placement (10 % overstretch, 20 s), damage was induced by balloon inflation (Abbott, Abbott Park, Il, USA) at the implantation site (15 % overstretch, 20 s). After employment of the stents, patency was confirmed angiographically. In case of ventricular fibrillation, Amiodaron (150 mg) was administered immediately followed by treatment with a manual external defibrillator until normal sinus rhythm was re-established. After confirmation of correct stent placement and patency, catheters were removed, the carotid artery was sutured and the wound was closed, and animals received buprenorphine (Temgesic, 0.02 mg/kg). Three months after stent implantation the same anesthetic protocol was applied for control angiography and after angiography, the animal was euthanized with a lethal dose of pentobarbital. The heart was rapidly excised, after which the stents were harvested and subsequently fixed.

### Sample processing

For quantitative morphometric analysis, stented arterial segments were fixed overnight in 4 % formaldehyde after excision and stored in 70 % ethanol. The segments were dehydrated in a graded series of acetone and embedded in resin (methyl methacrylate and butyl methacrylate, 1:1). Sections (7 μm) were cut with a rotary microtome (Leica) from the middle of the stent, after sawing the segment with a band saw (Exakt). Sections were attached to glass slides and dried overnight.

### Morphometric analysis

General histology and fibrin deposition was determined with hematoxylin and eosin (H&E) staining. Morphometric analysis and injury score according to Schwartz [19] were performed on sections stained with Lawson-van Gieson (LvG) staining. The lumen cross-sectional area, external elastic lamina area and internal elastic lamina (IEL) area corrected for strut holes were assessed with imaging software (Leica Qwin). The percentage of stenosis was calculated as [1-(lumen area/IEL area)]*100. Additionally, Masson’s Trichrome (MT) staining was performed to visualize the medial SMC layer and the adventitial collagen layer.

### Inflammation

Infiltration of inflammatory cells is an important factor in the evaluation of stent safety and is idiopathic for the porcine coronary artery model. Therefore, inflammation was scored in a blinded fashion by a pathologist as described before [20].

### Immunohistochemistry

Sections were stained immunohistochemically using antibodies against smooth muscle α-actin (αSMA, 1A4, DAKO), von Willebrand Factor (Millipore) and p27^Kip1^ (Abcam), followed by horseradish peroxidase (HRP)-conjugated goat anti-mouse antibodies (Southern Biotech) or poly HRP-anti-rabbit IgG (Immunologic, Duiven, the Netherlands) followed by 3,3-diaminobenzidine (DAB) substrate color development (Immunologic). p27^Kip1^ quantification was performed on 3 areas per stent section for all stents and expressed as positive area of the intima.

### Statistical analysis

Values are presented as mean ± SE. Mann–Whitney *U*-test was used for morphometry, inflammation score and IHC of 6-MP versus control group using Prism 5.03 (GraphPad Software, San Diego California, USA). Differences were considered statistically significant with *P* < 0.05.

## Results

### 6-MP release from stents

**Fig. 1:**
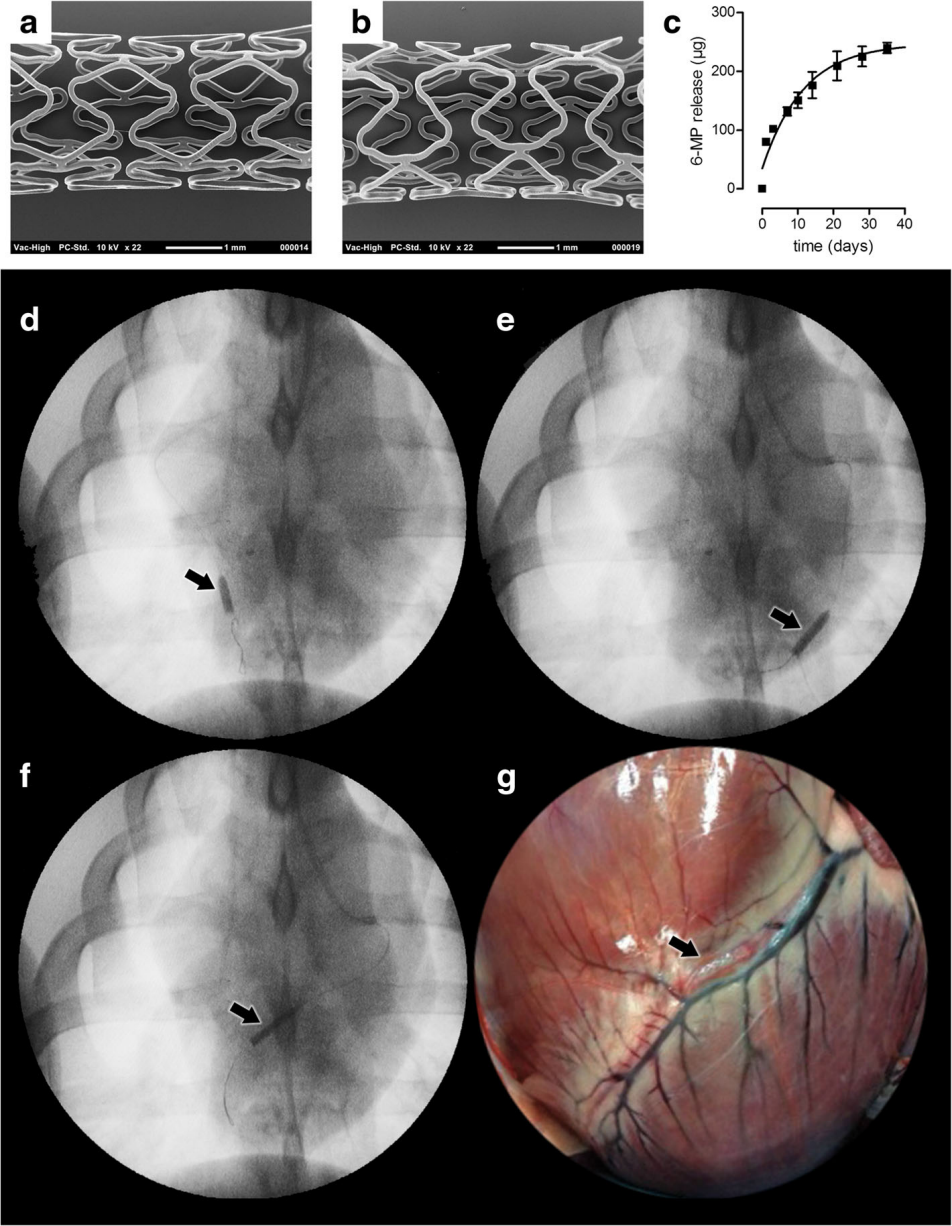
Characteristics and implantation of 6-MP DES in porcine coronary arteries. Polymer-only control stents (**a**) and stents loaded with 300 μg 6-MP (**b**) showed smooth coating surfaces by scanning electron microscopy. The cumulative release of 6-MP from the stents (**c**) was determined in vitro for up to 38 days (panels **a**-**c** adapted from [18]). After applying endothelial damage by balloon inflation, stents were deployed in the RCA (**d**), LCX (**e**) and LAD (**f**). Stent placement was followed by control angiography to ensure patency of the stented coronary artery. After 3 months, control angiography was repeated to reassess patency. After euthanasia, the heart was rapidly excised and the stents, visible as indicated (**g**), were harvested and fixed. Arrows indicate location of the stents

Characteristics of the stent coating, as well as release and stability of 6-MP were described before [17]. Briefly, coatings were evenly distributed over the stent and characterized by a smooth surface (Fig. 1a, b), which was not negatively affected by crimping, sterilization or expansion of the stent by the balloon catheter. 6-MP-eluting stents eluted 6-MP gradually according to first order release kinetics, delivering over 75 % of the drug within one month (Fig. 1c).

### Vessel wall characterization

**Fig. 2:**
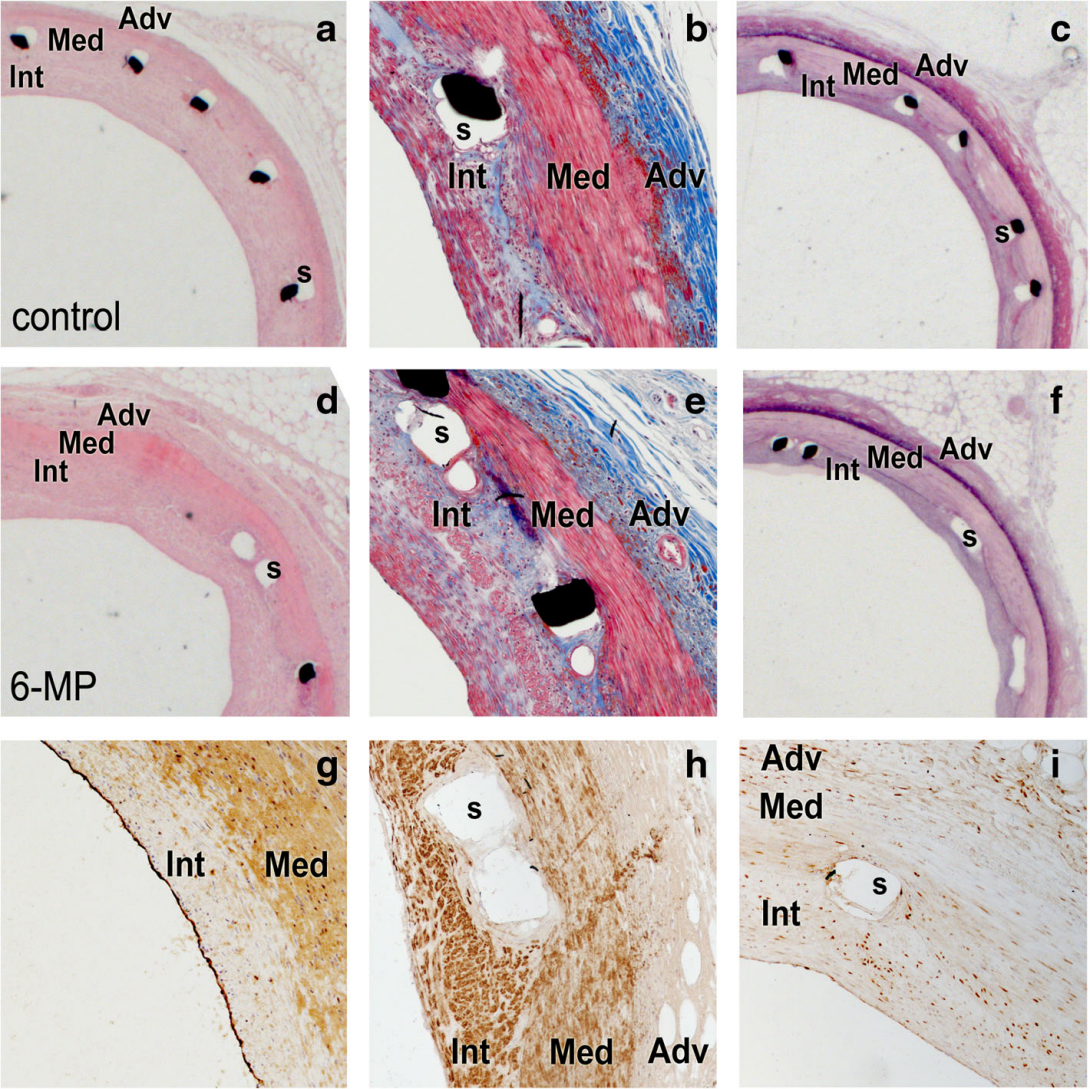
Characterization of stented coronary arteries 3 months after placement. Sections of arteries implanted with polymer-only stents (control) or 6-MP-eluting stents (6-MP) were stained with H&E to assess general histology of the stented vessel wall (**a**, **d**). Masson trichrome staining visualized the adventitia (Adv), media (Med) and intima (Int) of the vessel wall as well as strut holes (s) (**b**, **e**). Lawson-Van Gieson staining was applied to stain the internal and external elastic lamina and thus quantification of the different layers of the vessel wall (**c**, **f**), enabling morphometric analysis. Endothelial cell coverage of the vessel wall was demonstrated by immunohistochemical staining with an antibody against vWF (**g**). The media largely consists of circumferentially aligned SMCs, whereas SMCs in the intima are oriented longitudinally in the vessel wall, as shown by staining with an antibody directed against αSMA (**h**). Most cells positive for the cell-cycle inhibitor p27^kip1^ are localized in media and adventitia, whereas hardly any positive cells were found in the intima, indicating that especially in the intima cells are proliferating (**i**)

The 6-MP-eluting and polymer-only stents were implanted in porcine coronary arteries after local balloon injury to assess the effect of 6-MP on vascular lesion formation. Depending on the anatomical variation per animal, two or three stents were implanted in the RCA (Fig. 1d), LCX (Fig. 1e) and/or LAD (Fig. 1f). Three months after stent implantation, control angiography was performed under the same anesthetic protocol. Patency of all stents was visually confirmed by angiography directly after placement and after 3 months, before harvest of the stented segments (Fig. 1g). H&E staining on resin-embedded sections showed similar structure of the vessel wall between groups that received either polymer-only or 6-MP-eluting stents (Fig. 2a, d). No fibrin deposition was observed in the vessel wall. Masson Trichrome staining revealed similar organization of the vessel wall in both groups, and no difference in collagen deposition (Fig. 2b, e). To quantify different vascular layers, Lawson-van Gieson staining was performed visualizing the elastic laminae and connective tissue (Fig. 2c, f). To assess the presence of endothelial cell on the lesions, we performed a vWF staining and demonstrated complete coverage of the lesions with endothelial cells in all samples after 3 months (Fig. 2g). Stented vessel segments of both groups were stained with an antibody directed against the SMC marker αSMA; the adventitia is negative for this marker (Fig. 2h). In the media all SMCs are circumferentially aligned, whereas in the intima most cells stain positive and are longitudinally aligned SMCs. To obtain insight in the extent of quiescent and proliferating cells in the vessel wall, we performed an immunohistochemical staining for the cell cycle inhibitor p27^kip1^. p27^kip1^-positive cells were detected in all layers of the vessel wall in both groups, with relatively low expression in the intima (Fig. 2i). The latter indicates that as expected, most non-quiescent, proliferating cells are localized in the intima, whereas medial SMCs remain mostly quiescent.

### Morphometry

**Fig. 3:**
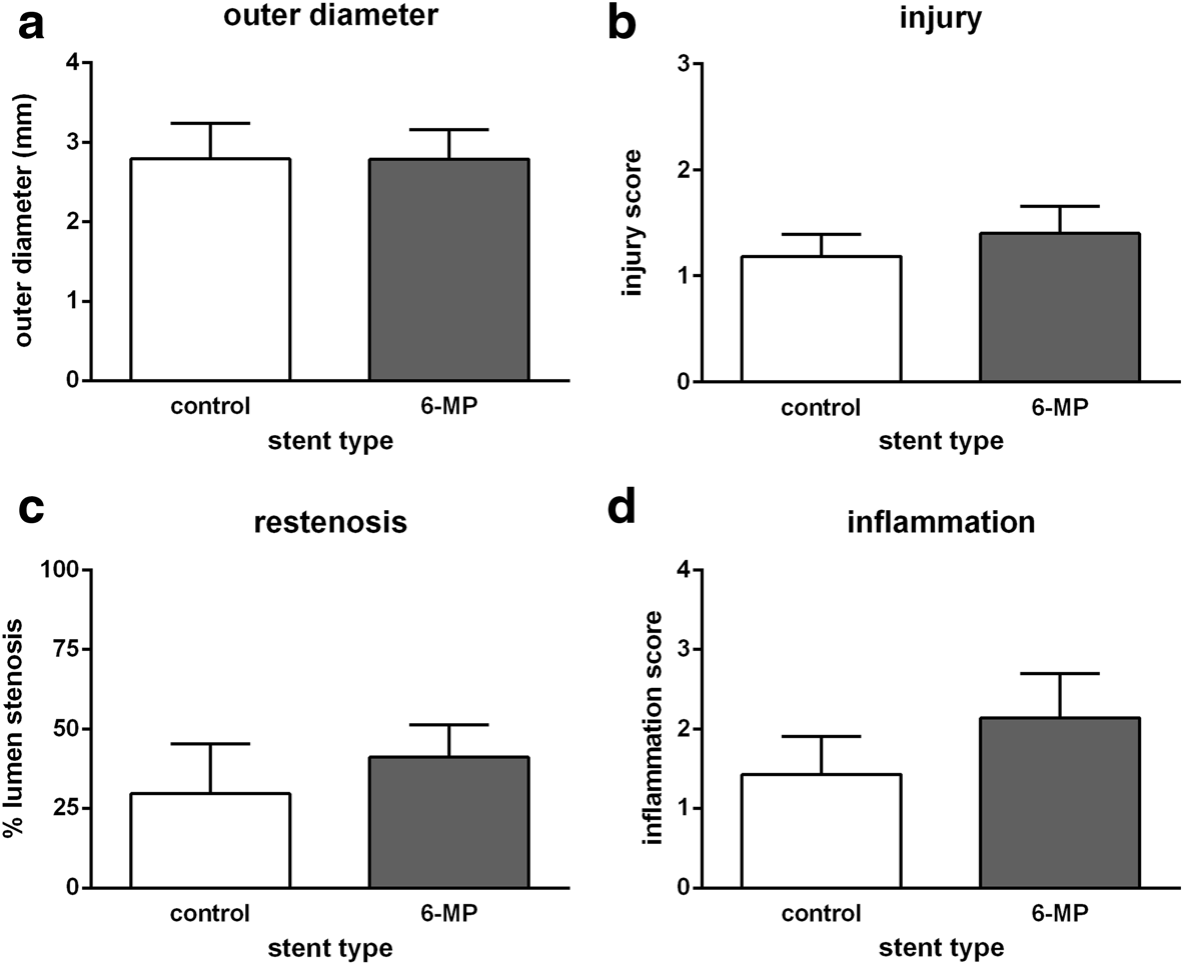
Morphometric analyses after 3 months of the stented coronary arteries. The outer diameter of the stented arteries (**a**) was similar within and between the groups, indicating high reproducibility of stent implantation and expansion. The injury score was low in all stents and similar between groups (**b**). Lumen stenosis (**c**) showed large variation within the groups and was similar between 6-MP and control stents. Inflammation score (**d**) was also similar between control and 6-MP. Bars depict mean values, error bars represent SE

Morphometric analyses revealed that stents were implanted reproducibly, as demonstrated by the similar outer diameter of the stents in both groups (Fig. 3a). In addition, injury score, a semi-quantitative measure for injury to the vessel wall, was low and similar in both groups (Fig. 3b). In-stent stenosis, the main outcome in this study, was not inhibited by 6-MP, as demonstrated by similar values for neointima thickness (Fig. 3c). Furthermore, thickness of media and adventitia was not affected by 6-MP (data not shown). In order to determine whether the vascular response was different between the different coronary arteries, lumen stenosis in LAD, LCX and RCA was compared. All coronary arteries displayed equal values, indicating that localization of the stents in the coronary arteries did not affect lesion size.

### Inflammation

Infiltration of inflammatory cells observed around the stent struts was predominantly symmetrical. Mostly, infiltrates were small and granulomas were present. Inflammation score was similar between 6-MP and control stents (Fig. 3d).

## Discussion

The primary goal during the original development of DES was to inhibit SMC proliferation, which has been achieved successfully and convincingly. Unfortunately, due to the drugs that were chosen for DES, the inhibition of cell growth is not limited to SMCs, but is accompanied by delayed endothelial recovery. Given that during stent placement the endothelial cell layer is severely injured, incomplete healing causes increased risk for thrombotic events within a year after stent placement, and even beyond that. As a consequence, patients need to adhere to dual anti-platelet therapy after DES placement for a prolonged period of time [3–5]. In order to combat this complication of current DES, our laboratory developed novel DES releasing 6-MP, a Nur77 agonist, which is known to have cell-type specific effects; 6-MP reduces SMC proliferation and the inflammatory response of macrophages, while promoting survival of ECs [6–17]. In rabbit iliac arteries, we demonstrated that 6-MP-eluting stents reduce stenosis and inflammation after 1 month, with effective endothelial coverage of the stent struts after 1 week [18]. Therefore, a similar effect was expected in the present study, since the 4-week rabbit iliac model and the 3-month porcine coronary model are well established in DES research [21, 22]. However, in the current study stents eluting 300 μg 6-MP did not display a reduction in intima formation or in macrophage infiltration in porcine coronary arteries after 3 months.

Since the novel 6-MP-eluting stents failed to reduce stenosis or inflammation in the 3-month pig model, we did not investigate endothelial cell coverage by scanning electron microscopy. The aim of the present study was to investigate the clinical potential of 6-MP-eluting stents. The lower 6-MP dose (100 μg) used in our previous study was not tested in the current model, since this had already been proven insufficiently effective in rabbits [18]. We did explore the possibility to increase the 6-MP dose on the stents. However, the enhanced coating volume necessary to load more drug (500 μg) resulted in stents with unfavourable mechanical characteristics (data not shown).

A limitation of the current study is the small number of observations. In combination with some variation in the outcome, it is hard to obtain statistical significance. The decision to use 3 different positions to implant the stents in order to reduce the number of animals needed, may have attributed to the variability. However, even with the current amount of observations there was no obvious trend towards improvement. Possibly, the inclusion of an additional time point, e.g. 28 days as often used in preclinical DES studies, might have revealed more information about the effect of 6-MP in the vessel wall. The narrow therapeutic window of 6-MP may explain the difference in outcome between the previous rabbit study and the current pig study. In vitro, 6-MP was shown to be effective at 10–50 μM to enhance endothelial cell survival, [23] whereas monocytes and macrophages respond to 50 μM 6-MP, and cultured SMCs require 25 μM 6-MP for an optimal growth inhibitory response [24, 25]. At higher concentrations 6-MP may lead to apoptosis, which is highly undesirable in the setting of an atherosclerotic, stented vessel wall. Cell death and fibrin deposition were not observed in the section, however. We may conclude that it is difficult to reach this therapeutic window for 6-MP in vivo in porcine coronary arteries. Even with controlled first-order release of a hydrophobic drug like 6-MP, penetration of the drug deep into the tissue may not have reached the required local concentration of 6-MP and optimal duration of the drug effect. A higher dose of 6-MP may be required to obtain the required 6-MP concentration in the vessel wall. Further development or refinement of the prototype is possible. Since higher drug concentrations are not easily obtained on the limited stent surface, a different release profile or duration may improve the outcome.

## Conclusions

In conclusion, 6-MP has previously been shown both in vitro and in vivo to potently inhibit SMC proliferation, reduce inflammation and promote endothelial cell survival. However, the 6-MP dose released from the currently developed 6-MP eluting stents was found to be insufficient to reduce stenosis or inflammation in porcine coronary arteries after three months.

## Abbreviations

6-MP: 6-mercaptopurine
DES: Drug-eluting stent
H&E: Hematoxylin and eosin
IEL: Internal elastic lamina
LAD: Left anterior decending artery
LCX: Left circumflex artery
LvG: Lawson van Gieson staining
MT: Masson’s trichrome staining
NR4A: Nuclear receptor 4A
RCA: Right coronary artery
SMC: Smooth muscle cell
αSMA: Smooth muscle α-actin
